# The complexities of integrating evidence-based preventative health into England’s NHS: lessons learnt from the case of PrEP

**DOI:** 10.1186/s12961-023-00998-4

**Published:** 2023-06-14

**Authors:** Tehseen Khan, Clare Coultas, Katharina Kieslich, Peter Littlejohns

**Affiliations:** 1 King’s College London, London, United Kingdom; 2 Department of Political Science, University of Vienna, Universitätsstr. 7, 1010 Vienna, Austria; 3 Spring Hill Practice, 57 Stamford Hill, London, N16 5SR United Kingdom; 4 School of Education, Communication and Society, King’s College London, Waterloo Bridge Wing, Waterloo Road, London, SE1 9NS United Kingdom; 5 Emeritus Professor of Public Health, Centre for Implementation Science, Institute of Psychiatry, Psychology and Neurosciences, King’s College London, 16 De Crespigny Park, London, SE5 8AB United Kingdom

**Keywords:** PrEP, Evidence-based policy, Preventative health, NHS commissioning, HIV

## Abstract

**Background:**

The integration of preventative health services into England’s National Health Service is one of the cornerstones of current health policy. This integration is primarily envisaged through the removal of legislation that blocks collaborations between NHS organisations, local government, and community groups.

**Aims and objectives:**

This paper aims to illustrate why these actions are insufficient through the case study of the PrEP judicial review.

**Methods:**

Through an interview study with 15 HIV experts (commissioners, activists, clinicians, and national health body representatives), we explore the means by which the HIV prevention agenda was actively blocked, when NHS England denied responsibility for funding the clinically effective HIV pre-exposure prophylaxis (PrEP) drug in 2016, a case that led to judicial review. We draw on Wu et al.’s (Policy Soc 34:165–171, 2016) conceptual framing of ‘policy capacity’ in undertaking this analysis.

**Results:**

The analyses highlight three main barriers to collaborating around evidence-based preventative health which indicate three main competence/capability issues in regard to policy capacity: latent stigma of ‘lifestyle conditions’ (individual-analytical capacity); the invisibility of prevention in the fragmented health and social care landscape related to issues of evidence generation and sharing, and public mobilisation (organizational-operational capacity); and institutional politics and distrust (systemic-political capacity).

**Discussion and conclusion:**

We suggest that the findings hold implications for other ‘lifestyle’ conditions that are tackled through interventions funded by multiple healthcare bodies. We extend the discussion beyond the ‘policy capacity and capabilities’ approach to connect with a wider range of insights from the policy sciences, aimed at considering the range of actions needed for limiting the potential of commissioners to ‘pass the buck’ in regard to evidence-based preventative health.

## Background

In 2016, the National Health Service England’s (NHSE’s) refusal to commission pre-exposure prophylaxis (PrEP), a clinically effective drug for treating human immunodeficiency virus (HIV), led to the National AIDS Trust seeking a judicial review, which they won. Routine access to PrEP is currently being rolled out as part of a combination approach to HIV prevention in England, commissioned by local authorities, with specialist sexual health services targeting groups at high risk of contracting HIV [1]. Whilst PrEP is of course a unique case, we propose that much can be learnt from it regarding the complexities of commissioning evidence-based preventative health in England. The NHS Five-Year Forward View states that “the sustainability of the NHS, and the economic prosperity of Britain all now depend on a radical upgrade in prevention and public health” [2], p. 3. Moves towards more ‘integrated care’ are being operationalised through changing ‘primary legislation’ so that NHS organisations, local government, and community groups can collaborate more easily [3–6]. However, our interview study with different stakeholder groups undertaken in 2017, implicates barriers that go beyond the removal of legislation, pertaining to the ways in which evidence is [not] generated and [not] used by health practitioners and policymakers.

### Commissioning prevention interventions in England

The Health and Social Care Act of 2012 (HSCA 2012) moved the prime responsibility for commissioning prevention interventions for Public Health in England from the NHS to Local Authorities. However, there remains considerable ambiguity about the relative roles and responsibilities of key institutions. For example, in the case of sexual health, local authorities are responsible for commissioning sexual health services (e.g. prevention and treatment of sexually transmitted infections, contraception, and HIV prevention and testing); NHS England (NHSE) commissions HIV treatments as a ‘specially commissioned’ service, along with vaccines (e.g. HPV, hepatitis), and any sexual health services provided through General Practitioner contracts (e.g. cervical screening); clinical commissioning groups (CCGs) commission abortion services; whilst Public Health England (PHE) held an advisory role in regard to strategy and disease surveillance (the newly formed Health Security Agency now holds this function). This fragmentation of responsibility, together with widespread cuts to local authority budgets (up to 50%), and a reduction in centralised ring-fenced public health budgets, has drawn concerns regarding the overall decline and postcode lottery of sexual health services [7, 8]. Current health policy seeks to reverse this trend, ostensibly heralding the end of the 40-year experiment with the purchaser-provider split and quasi-markets in the NHS, with improvements in NHS action on health prevention and health inequalities being a core rationale for ‘integrated care’ [3].

### The PrEP case

In recent years there have been significant developments in HIV prevention strategies of which PrEP is one. Different from Treatment as Prevention (TASP) and post-exposure prophylaxis (PEP), PrEP involves using Truvada, an anti-retroviral treatment, *prior* to HIV exposure. The PROUD and IPERGAY studies of its use in United Kingdom and France indicate up to an 86% risk reduction in HIV incidence [9, 10], and whilst there remains a lack of consensus, many believe PrEP has played an important role in the dramatic and continuing declines in HIV incidence amongst men who have sex with men (MSM) in the United Kingdom since 2016 [11]. Part of the ambiguity around its contribution to these decreasing numbers of new HIV diagnoses is that individuals have been independently purchasing PrEP online since October 2015, meaning that the total numbers using it remain largely unknown [11].

Initial indications were that NHSE was preparing to commission PrEP as a specialised service: in September 2014 NHSE set-up the HIV Clinical Reference Group to outline the policy for PrEP; and in 2016 it commended the encouraging findings of the PROUD study and identified itself as the responsible commissioner for the drug, whilst local authorities would carry the service costs for providing PrEP through sexual health clinics [12–14]. However, in March 2016, NHSE issued a statement on its website claiming that due to external legal advice, it did not have the power to commission PrEP, drawing on the HSCA 2012 that identifies local authorities as the responsible commissioner for prevention services [12]. The National AIDS Trust sought a judicial review to challenge this decision, which it won in July 2016, with the judges rejecting the argument that NHSE has no prevention responsibilities [15]. NHSE then appealed this decision in August 2016 and published a statement on its website claiming that the main group to benefit from PrEP was “men who have high risk condomless sex with multiple male partners” [13], drawing controversy for its homophobic connotations and stigmatising representation of HIV risk as a ‘lifestyle’ [16]. The appeal was lost, with the judges considering PrEP as a treatment rather than prevention, rendering the argument that NHSE cannot commission it redundant. Nevertheless, whilst ruling that NHSE does have the power to commission PrEP, the Court of Appeal did not oblige it to do so.

Internal NHSE documents (sourced through a freedom of information request) reveal that questions remained over the method by which the cost-effectiveness of PrEP was calculated initially: they had looked at the per person cost of the drug rather than the number of people that needed the drug in order to prevent one infection (estimated to be 36) which would have ruled PrEP out of being commissioned [14]. Concerns were therefore raised about the fairness of comparing preventative and other treatments on a like for like basis [14]. In December 2016 NHSE announced that it would fund a £10 million PrEP impact trial over 3 years making PrEP available to 10 000 participants, that was then expanded to reach 26 000 individuals by 2020 [4, 5]. The trial was controversial however: stories surfaced describing people being turned away from clinics where trial places were full [17], and analyses of the trial protocol highlighted its use as a means of rationing access to PrEP as opposed to answering a scientific question, for instance related to effectiveness [18].

## Methods

The aim of the study was to understand the factors influencing NHSE’s decision-making during the PrEP assessment process and to gain insight into stakeholder perceptions of why a judicial review was required. We expanded the scope of the study drawing on Wu et al.’s [19] conceptualisation of ‘policy capacity’ [19] which highlights “the set of skills and resources—or competencies and capabilities—necessary to perform policy functions… categorized into three types: analytical, operational, and systemic… [which operate] at three different levels—individual, organizational, and systemic” (p. 166). This conceptual framework enabled us to identify the specific aspects and dynamics which act against the incorporation of preventative health into England’s NHS.

Participants were selected through purposive and snowball sampling, and between June and July 2017, TK carried out semi-structured interviews with 15 stakeholders who were either involved with, or affected by, the commissioning of sexual health services and PrEP: three PrEP activists; three sexual health clinicians; three commissioners; and six national health body representatives (who were privy to the decision-making in regard to PrEP, and worked with decision-makers, however were not decision-makers themselves). The study was designed by TK, KK, and PL, and questions focussed on understanding the participant’s role and relation to the commissioning of sexual health services, their understanding of the factors that led to NHSE’s decision not to commission PrEP, their experience of the decision-making process, and perspectives on the main lessons learnt from the PrEP case. The interviews were conducted by TK at a venue of the person’s choosing, and lasted an average of 47 min. Consent for recording and transcribing the interviews was obtained from 14 participants on the condition that any reference to person, place, and organisation be removed. One participant declined audio-recording but agreed to the researcher taking written notes. The study received ethical approval by the King’s College London Research Ethics Committee (reference: LRU-16/17-4567).

The sample size of the study is small, but this is not uncommon in qualitative studies that seek to investigate the reasons or meaning for certain events. The focus of the study was to provide initial exploratory insights on some of the reasons why NHSE decided not to commission PrEP even though it had initially suggested it would, and how this made a judicial review necessary. Thematic saturation was reached in the interviews, and in the discussion section of this paper we illustrate that the initial failure to commission PrEP has implications and meaning for future case of commissioning preventative services and interventions.

A thematic approach was used for data analysis [20]: codes were inductively generated by TK, CC, and KK, with themes generated through discussions between TK, CC, KK, and PL, in accordance with Wu et al.’s [19] ‘policy capacity’ framework. A secondary analysis was also undertaken, exploring patterns related to participants’ roles. The COREQ guidelines [21] for reporting qualitative research were followed.

## Results

Across the stakeholder groups, participants cited the cost of PrEP as the underlying reason behind the NHSE's decision. The majority also viewed the HSCA 2012 negatively with respect to the de-prioritisation of public health. In addition to these, three main barriers to collaborating around preventative health were identified, which draw attention to different competences and capabilities in regard to policy capacity: latent stigma of ‘lifestyle’ conditions—individual-analytical capacity; the invisibility of prevention in the fragmented health and social care landscape—organizational-operational capacity; and institutional politics and distrust—systemic-political capacity.

### Latent stigma of ‘lifestyle’ conditions: individual-analytical capacity

Wu et al.’s [19] articulation of individual-analytical capacity pertains mainly to people’s “ability to access and apply technical and scientific knowledge and analytical techniques” (p. 168), and we suggest that the present study indicates an important expansion to this definition—namely, the role that values play in health policy and systems decision-making [22]. Whilst the homophobia surrounding the PrEP case was discussed in many of the interviews, the activists and two of the clinicians also highlighted issues of more generalised latent stigma within the health system. For the activists, these discussions centred on how current funding structures are driving inequalities:

“Why is it… that clinical treatment… [for] sexual health and drug services are not within the NHS? Is this an accident that the two bits of acute clinical care, which involves some of the most marginalized and stigmatized people are being taken out of National Health Service including the budgetary protections? No it's not” (Activist.10).

Two clinicians identified this latent stigma as more broadly being against ‘lifestyle’ choices (discussed shortly), and in fact three of the other study participants (all of whom still hold active clinician roles) expressed such stigma:

“I’m just a bit cynical, are people just wanting to take it [PrEP] to have more risky sex? Yes, in 9 out of 10 cases” (Clinician.12)*.*

“Obesity is immoral when so much of the world is starving… prevention without medication is better. I would promote safe sex, relationships, love… I would teach philosophy about how to live a good life. A life that is stable, secure and not harmful to yourself or others” (Commissioner.6).

“If we know there is a pill that can fix part of what I’ve self-caused, let me take my self-cause to the next level up, which isn’t fixable… the most cost-effective way to stop HIV is to use barrier contraception” (Nat.Rep.8).

As the two clinicians emphasised, this representation of PrEP as “a lifestyle drug for gay men” not only obfuscates the social determinants of health, and too, diversities within these, excluding other at-risk groups (e.g. African communities) from accessing PrEP (Clinician.5), it also neglects a fundamental principle in public health:

“PrEP is a hook that allows us to engage with some of the most important [at-risk hard-to-reach] patients… it engages… [them] in care… [to get their prescription they must] see us every three months, we’ll do an STI screen… if they’ve got issues with chemsex and drug addiction we can start those conversations and deliver that counselling… [and] behavioural interventions… [long-term] it’s cost-effective” (Clinician.9).

One of the national health body representatives described the transfer of sexual health to local authorities as nonsensical owing to how medicalised the field is, suggesting that it must have been a “back of the envelope solution late at night when they got around to thinking, ‘god none of us thought of that we better put it somewhere, put it with local’—…creating all sorts of issues” (Nat.Rep.15). In contrast, the national health representative who viewed PrEP as a risk-enabling lifestyle drug argued that GPs are intentionally identified as the incorrect provider for sexual health: “I know very few adults bold enough to be able to go to a GP and have a conversation and say I’m about to have unprotected sex… It’s going to be an extraordinarily difficult conversation… Because we’d have to counsel them against it” (Nat.Rep.8).

Of note, the integration of HIV prevention into GP practices was exactly what the activists were arguing for: “It needs to be available in GP surgeries for new registrations and train GPs to spot what could be seroconversion illnesses so they could suggest to their patient ‘oh, have you considered a HIV test’ so it becomes more routine. That requires coordination” (Activist.3).

### The invisibility of prevention in the fragmented health and social care landscape: organizational-operational capacity

Wu et al. [19] describe organizational-operational capacity as pertaining to the organization of public agencies and the political-institutional environment in which they operate. The interviews highlighted how the fragmented health and social care landscape in England (stemming from the HSCA 2012) facilitates the invisibility of preventive health. Two commissioners and four national health representatives discussed how there are frequently confusions over who funds what, that in the current climate of significant budget cuts, often results in time wasted owing to lengthy negotiations, and NHSE was mentioned specifically as using tactics of avoidance:

“NHSE are very clearly responsible for commissioning the cervical screening programme, but they have not always taken that responsibility seriously. After 2013, NHSE… [detailed] certain clinics as places where women could go for their smears but NHSE were not actually funding these clinics. Local authorities were picking up the bills… I attend a lot of meetings and for the last 18 months [of budget cuts] NHSE have not been present in any of them. I think that’s disgraceful… they don’t attend due to fear of being criticised” (Commissioner.2).

The activists remarked how this avoidance and delay so often goes unnoticed owing to how difficult it is to mobilise people around evidence-based prevention, “people [don’t want] to see themselves as potential patients” (Activist.1). In this way, the PrEP case is special in that an active community was waiting for NHSE’s consultation, “there was a vacuum, there was no handover” (Activist.10), and when it didn’t happen, “we sent out a tweet, #whereisPrEP… and within 24 h it was picked up by others in the sector” (Activist.1). Yet the clinicians and commissioners identified how the system overall was not built to incentivise prevention:

“If I, [a local authority commissioner] spend money on PrEP, they [the NHS] get the benefit” (Commissioner.7).

“you're a respiratory clinic dealing with COPD, there’s no real driver to help you support smoking cessation … [or] for you to do partner notification and support people around PrEP and PEP. You do it, but there’s no real penalty for not doing it, or doing it badly…you're not paid on the number of HIVs you prevented… that’s the problem with prevention, it’s hard to prove a negative” (Clinician.9).

“PrEP is cost saving, but this is over a long period of time and commissioners and the government aren’t interested in the long-term” (Clinician.5).

This last point was discussed by a number of clinicians, commissioners, and national health body representatives as an issue of evidence, particularly in regard to the type of evidence collected, but also difficulties in accessing evidence:

“the movement of the Public Health grant into local government could have been an amazing move… if the government said ‘look, we’re going to ring fence it for now but actually we’re going to… [generate evidence on] return of investment and we’re going to start investing in those things… but instead it was, ‘move it over there and, oh, we’re going to cut it now because it’s a Cinderella service’” (Clinician.9).

“We’re dealing with such acute budget cuts [in local authorities]. They’re re-procuring school services, sexual health services… [and] private companies, there’s always more risk. They won’t do anything that’s not in the contract” (Nat.Rep.11).

“Our biggest danger in health monitoring is privatisation… [and] the loss[/inaccessibility] of data” (Nat.Rep.4).

“there was not a high level of certainty that PrEP at the price would be cost-effective… how many GUM clinic attendees would meet the high-risk eligibility criteria? Unknown… [and] the additional service costs… [depend] on the nature of the contract between the providers and the commissioners… [and] there’s no national information pool of how many block contracts there are or what’s in the block contract” (Nat.Rep.15).

### Institutional politics and distrust: systemic-political capacity

Two commissioners and two national health body representatives spoke at length about ‘top-down’ governmental control over commissioning and publications of evidence, that are enabled by the currently fragmented system, and which contradicts the language of localism in the NHS long-term plan:

“The creation of NHSE was an opportunity to create independence from government but in reality they have just become the comms department for the Department of Health… The government has been able to stick with a national message to say they are protecting NHS budgets but have cut health care budgets… Everyone worries about local authorities raiding public health budgets but in actual fact, the robber in the wing is national government… [so maybe the decision not to fund PrEP] was political, to rattle the cage of the Secretary of State… they picked something particularly topical and sensitive to push back on… [to] get government to cough up more cash?” (Commissioner.2).

“this is classic central government dividing and ruling and reducing funds from the top. Local residents then don’t look at central government but criticise NHSE.” (Commissioner.7).

“We’ve been wanting to talk about the fall in HIV in London but we’ve not been allowed to publish it for seven weeks… PHE was supposed to be established as an expert body to assist the government but not manoeuvred by government but… I think we’re under more scrutiny by government than we ever were as the Health Protection Agency. We don’t have our own website, all of the information is from the government” (Nat.Rep.4).

These dynamics speak to what Wu et al. [19] describe as ‘systemic-political capacity’, namely, the environment which frames and steers all other aspects of policy capacity, intrinsically connected to issues of [dis]trust. All of the activists, commissioners, and clinicians discussed how the lack of transparency in NHSE’s decision not to fund PrEP, had only exacerbated their own and others’ distrust in NHSE, with many describing it as a ‘cover up’:

“The right people [were] in the room [for the Clinical Reference Group]… [however suddenly] someone very important… intervened and shut the thing down. It certainly wasn’t transparent, it certainly wasn’t a matter of ‘this is what we think, we’re worried, do you have a different view?’, there was no discussion” (Activist.10).

“my colleagues said that they dialled in… expecting a wrap up call… the last step in the Clinical Reference Group. The call instead is NHSE saying they were not the responsible commissioner and it is up to local authorities to commission... Everyone was completely taken by surprise, people who had worked with the process and had given their professional time and energy to it were staggered” (Commissioner.2).

“if the government had turned around and said ‘do you know what, we’re going to wait until it’s generic and then we’re going to fund it’ and made that decision based around cost, I think that would have been far more honest…” (Clinician.9).

Yet two of the national health body representatives remarked on how these factors were not a concern to PHE and NHSE, in that “the turnover of staff… [means] in three years time, no one would even remember it [the court case]” (Nat.Rep.4).

## Discussion

We used insights from Wu et al.’s [19] conceptual framework for policy capabilities and competences to guide our analysis because at the beginning of the study we conceived of the PrEP case as an example of a policy failure, or at least a misalignment of intended goals and actual outcomes of preventative health policy in England. The interview data, however, shows that the PrEP case had important lessons beyond policy capabilities, and that the findings can be interpreted using a wider range of insights from the policy sciences than the literature on policy capabilities. In this discussion we offer a critique of the policy competences and capabilities framework, and a brief elaboration of other theoretical approaches that may explain the intricacies of the PrEP case in the England’s NHS.

### Recognising values in individual-analytical capacities in health policy and decision-making

The policy capabilities framework by Wu et al. [19] rightly points out that the analytical capacity of individuals who are in charge of formulating, implementing or evaluating policy is an important factor in how evidence is applied and policy is made. Wu et al. [19, p. 167] state that the policy capacity of these individuals: “[…] is determined by their knowledge about policy processes, skills in policy analysis and evaluation, managerial expertise and political judgement”. The framework, however, fails to acknowledge that individuals are also shaped by context-specific factors such as their beliefs and values. The literature on advocacy coalitions [23] and policy paradigms [24], broadly situated within the ideational turn of public policy [25, 26], shows how policy actors are shaped by the values they hold and which they bring to the policy tables of this world. This explains, for example, why the views that reflect latent stigma about the potential patient group who would be eligible for PrEP were so manifold in the interview data. Stigma and stereotypes reflect personal beliefs or values that are influenced by political and societal culture, and which can become normalised and persist latently in professions and institutional practices. They take time, as well as conscious political and societal efforts, to reverse and to call out. Issues of potential social acceptability bias in the interviews prevent us from drawing conclusions about the extent of the problem of stigma or about its ultimate influence on NHSE’s decision, but the frequency with which discriminatory opinions were voiced, suggests we can assume that it had at least a part to play in how the PrEP case played out.

The issues of stereotyping, stigmatization, and the role of beliefs and values therein offer insights for public health and prevention beyond the PrEP case. They are related to the longstanding debates in health and public health about the role that personal responsibility does, or should, have when making decisions on which services to fund, and for whom (e.g. [27–29]). A popular example that is often given is whether a person who engages in risky behaviour such as bungee jumping should have to pay for their care themselves if they ever need to be treated for injuries as a result of such activities. Similarly, in public health the debate about ‘lifestyle choices’ is over the extent to which campaigns for healthy eating, smoking cessation or alcohol consumption reduction should include appeals to personal responsibility [29]. These tensions and debates are clearly reflected in our interview data, for example, when one clinician talked about their concern that the availability of PrEP would just increase unsafe sex practices which are framed as lacking in personal responsibility. Research also indicates that medications for alcohol relapse prevention are underutilized in England despite NICE guidelines and the evidence-base for their use, and that dispensing ratios can be even lower for socially disadvantaged groups [30].

The issue of stigma highlights the need for both public consultation and clinician engagement regarding how support for ‘lifestyle’ conditions is provided. The strong discord between activists’ desires that sexual health care be integrated into GP practices, and some of the clinician views that people would not talk about these issues to their GP, needs to be examined. A study in Australia highlights the politics inherent to how different HIV experts are engaging with PrEP, illustrating the ways in which they ‘assemble’ evidence in particular ways for particular ends, forming three main stances: concerned/alarmed (i.e. viewing that PrEP will reduce condom use and perpetuate STIs and antibiotic resistance); neutral/normalising (i.e. that PrEP is just a new tool that needs to be incorporated into practice and will have little effect on condom use and STI prevalence); and optimistic (i.e. viewing PrEP as holding the potential to encourage more engagement and could lead to reduced STI rates—[31]). In England’s healthcare context the issue of differing political and social values goes beyond clinicians. The political ideology of local authorities, or more specifically, the dominant views held about the causes of ‘lifestyle’ conditions—broadly distinguished as individual responsibility versus social determinants—can influence the kinds of interventions that are prioritised [32]. For instance, two boroughs exited the London HIV Prevention Programme despite the evidence of positive outcomes [48]. Likewise there have been significant differences in local authority provision of nicotine replacement therapies (NRT) in smoking cessation programmes [33], and the first and only clinic in England to provide heroin assisted treatment (HAT), which has a robust international evidence base [34], has recently been closed owing to a change in local government [35].

### The need for organizational-operational and -political capacities for preventative health policy

Centralised preventative evidence-based strategies for ‘lifestyle’ conditions are needed as a matter of urgency because risk is not contained within boroughs, therefore neither can care be. The public health approach described by clinician 9, in which care is seen as a means of relationship-building with hard-to-reach groups and enabling long-term savings on the system, indicates a clear role and value for healthcare practitioners that transcends politics and place. The PrEP case tells a cautionary tale of isolated organisational decision-making in times of financial austerity, and highlights the ongoing complexities of building organizational-operational capacity for preventative commissioning and care in and amongst the lasting effects of fragmentation in England’s health system. This therefore holds implications in terms of organizational-political capacity, described by Wu et al. [19] as relating to “developing learning relationships with governance partners and the public” (p. 169). Several interview respondents told us about their shock at NHSE’s U-turn over the commissioning of PrEP, and this was perceived to have taken place in a manner that was not transparent. The fact that difficult decisions need to be made, and priorities set, when budgets are tight is widely acknowledged in the literature on health priority setting (e.g. [36–38]). This literature also suggests that difficult decisions are more likely to be accepted by the public, patients and other stakeholders if the decision-makers are transparent about how and why decisions were made [39]. The latter was not the case in NHSE’s handling of PrEP. If, as some of the interviewees suggested, NHSE really saw a chance to ‘pass the buck’ to another entity, in this case the local authorities in England, and thereby avoid the costs that come with commissioning a new drug or intervention, then NHSE would have done better to say so openly. The discrepancy between cost effectiveness and budget impact of an intervention or treatment is not new, not least because some of the recommendations that the National Institute for Health and Care Excellence has made in the past have had severe impacts on the health care budget [40]. NHSE could have started a debate on the budget impact of PrEP if its concern was costs. Instead, by being opaque about the reasons for its U-turn, it caused itself reputational damage resulting in distrust from the organisations and people it is supposed to work with and for, in the provision of preventative services in the future. In other words, NHSE’s U-turn and the successful judicial review likely has had negative effects on its organisational-political capacity to build relationships and collaborations for preventative health.

The developments and failures in the case of PrEP did not happen in a political vacuum, but as a direct result of the HSCA 2012 that divided responsibility for prevention and public health between NHSE and local authorities without providing specifics of what this would look like. Additionally, the organisational-political competencies and capabilities of Public Health England, another key player in prevention, were unclear. While this confusion about roles and mandates contributes to what happened in the case of PrEP, it does not justify what happened, not least because it is well documented that organisational chaos has potential negative effects on the quality of services. In his interim report evaluating screening programmes following national lapses in breast and cervical cancer screening, Sir Mike Richards [41] posed the question “who is in charge… [and] The answer is not obvious” (p. 3). The issue of fragmentation is longstanding in England’s health system, and particularly so regarding sexual health [42, 43].

### The importance of building systemic capacities in the wake of fragmentation

Current efforts at integration need to go beyond simply removing legal and statutory barriers. As highlighted in the case of PrEP, efforts need to be put towards building trust—systemic-political capacity—(with)in England’s health system in the wake of longstanding fragmentation. In regard to prevention specifically, funding streams will always be a contention in relationship-building owing to its ‘invisibility’ in the fragmented health and social care landscape. Historically, it has been easier to protect health budgets than local authority social care budgets and this is likely to be the same in the future. There is increasing evidence that the NHS is becoming frustrated by council decisions to disinvest from public health and are seeking to take responsibility back for these functions [44]. However, beyond challenges associated with aligning funding streams, the interviews highlight the urgent need for a re-evaluation of evidence-making in regard to the specific needs of prevention efforts, and how organizational-operational capacity might be bolstered by investments in ‘systemic-analytical capacity’ [19], namely how data is collected and disseminated. For instance, why is certain information, essential for more long-term forecasting, not being collected, and why is it that data access is not being negotiated with private contractors? We appreciate that evidence-based public health is complex owing to the breadth of evidence forms, the multiple levels of explanation, and the length of causal chains [45]. Yet without investment and strategic leadership focussed on integrating evidence streams for understanding prevention, its cost-saving potentials will remain largely unknown.

The interviews therefore highlight how barriers to integration lie not only in horizontal relationships between institutions, but also vertically between centralised government and their non-departmental public bodies. The goal of the HSCA 2012 was to separate the daily running of the NHS from the Department of Health and Social Care but observers have voiced that this separation was unlikely to be sustainable or feasible. The interviews illustrate how transparency and accountability remain a core aspect of overall distrust in the system. There are clearly wider agendas regarding the current restructurings of public services [46], centred on rolling back the state. Yet as seen in the case of PrEP, patient groups and the public still have some power of influence. The question remains as to how this power will, and can be, exercised in the newly established integrated care systems.

## Conclusion

The next years will be crucial in determining whether the NHS can embrace prevention in a meaningful way. Rather than ‘passing the buck’ in cases such as PrEP, NHSE needs to show strong leadership in strategizing for prevention. This demands action that goes beyond removing legislation, with attention being put towards: the ongoing challenges of demarcating exactly where prevention ends and treatment begins, and the implications of this for clinician and organisational roles and responsibilities; the need for a redesign of evidence systems and pathways; and developing new ways of working beyond the NHS’ ‘traditional top-down command and control style’ [47]. Despite the rhetoric in the Five-Year Forward View, the PrEP case suggests that the NHS still struggles to address questions of prevention in a way that is fit for a twenty-first century health service. To do so requires not just public funds or new technologies, but also the political and organisational will to move beyond how things have been done in the past.

## Data Availability

The data that support the findings of this study are available on request from the corresponding author [CC]. The data are not publicly available due to them containing information that could compromise research participant privacy/consent.
